# Chromosomal 16p microdeletion in Rubinstein-Taybi syndrome detected by oligonucleotide-based array comparative genomic hybridization: a case report

**DOI:** 10.1186/1752-1947-6-30

**Published:** 2012-01-23

**Authors:** Md A Mohd Fadley, Azli Ismail, Thong Meow Keong, Narazah Mohd Yusoff, Zubaidah Zakaria

**Affiliations:** 1 Unit of Hematology, Cancer Research Center, Institute for Medical Research, 50588 Kuala Lumpur, Malaysia; 2 Department of Pediatrics, Faculty of Medicine, University of Malaya, 50603 Kuala Lumpur, Malaysia; 3 Advance Medical and Dental Institute, Universiti Sains Malaysia, 13200 Kepala Batas, Pulau Pinang, Malaysia

## Abstract

**Introduction:**

Chromosomal aberrations of chromosome 16 are uncommon and submicroscopic deletions have rarely been reported. At present, a cytogenetic or molecular abnormality can only be detected in 55% of Rubinstein-Taybi syndrome patients, leaving the diagnosis in 45% of patients to rest on clinical features only. Interestingly, this microdeletion of 16 p13.3 was found in a young child with an unexplained syndromic condition due to an indistinct etiological diagnosis. To the best of our knowledge, no evidence of a microdeletion of 16 p13.3 with contiguous gene deletion, comprising cyclic adenosine monophosphate-response element-binding protein and tumor necrosis factor receptor-associated protein 1 genes, has been described in typical Rubinstein-Taybi syndrome.

**Case presentation:**

We present the case of a three-year-old Malaysian Chinese girl with a *de novo *microdeletion on the short arm of chromosome 16, identified by oligonucleotide array-based comparative genomic hybridization. Our patient showed mild to moderate global developmental delay, facial dysmorphism, bilateral broad thumbs and great toes, a moderate size atrial septal defect, hypotonia and feeding difficulties. A routine chromosome analysis on 20 metaphase cells showed a normal 46, XX karyotype. Further investigation by high resolution array-based comparative genomic hybridization revealed a 120 kb microdeletion on chromosomal band 16 p13.3.

**Conclusion:**

A mutation or abnormality in the cyclic adenosine monophosphate-response element-binding protein has previously been determined as a cause of Rubinstein-Taybi syndrome. However, microdeletion of 16 p13.3 comprising cyclic adenosine monophosphate-response element-binding protein and tumor necrosis factor receptor-associated protein 1 genes is a rare scenario in the pathogenesis of Rubinstein-Taybi syndrome. Additionally, due to insufficient coverage of the human genome by conventional techniques, clinically significant genomic imbalances may be undetected in unexplained syndromic conditions of young children. This case report demonstrates the ability of array-based comparative genomic hybridization to offer a genome-wide analysis at high resolution and provide information directly linked to the physical and genetic maps of the human genome. This will contribute to more accurate genetic counseling and provide further insight into the syndrome.

## Introduction

Rubinstein-Taybi syndrome (RSTS) is a well delineated multiple congenital anomaly syndrome characterized by mental retardation, broad thumbs and toes, short stature and specific facial features [1]. The syndrome is, at least in part, caused by a microdeletion at chromosome 16 p13.3 or by mutations in the gene for the cyclic adenosine monophosphate-response element-binding protein (*Crebbp*), which is located at 16 p13.3 [2]. The occurrence is generally sporadic and birth prevalence is one in 100, 000 to 125, 000 [2]. About 55% of patients have cytogenetic or molecular abnormalities in the *Crebbp *or E1A binding protein p300 (*Ep300*) gene, leaving the diagnosis in 45% of patients to rest on clinical features only [3]. However, due to the absence of a distinct clinical presentation and the limitation of routine chromosomal analysis techniques, definitive diagnosis in younger children is difficult.

Conventional cytogenetic analysis of patients with syndromic features has largely reported findings of a normal karyotype. This necessitates the adoption of a more sensitive method to detect the occurrence of any submicroscopic chromosomal aberration. Microarray-based comparative genomic hybridization (array-CGH) is a high-throughput technique that offers rapid genome-wide analysis at high resolution. Array-CGH is designed to detect not only genome-wide chromosomal and segmental deoxyribonucleic acid (DNA) copy number changes, but also map and measure the regions of amplification, which may be associated with a wide range of diseases, from developmental disorders to cancer. The use of current array-CGH technology enables the detection of submicroscopic chromosomal aberrations at multiple loci and localization of disease gene regions for subsequent candidate gene identification.

## Case presentation

**Figure 1:**
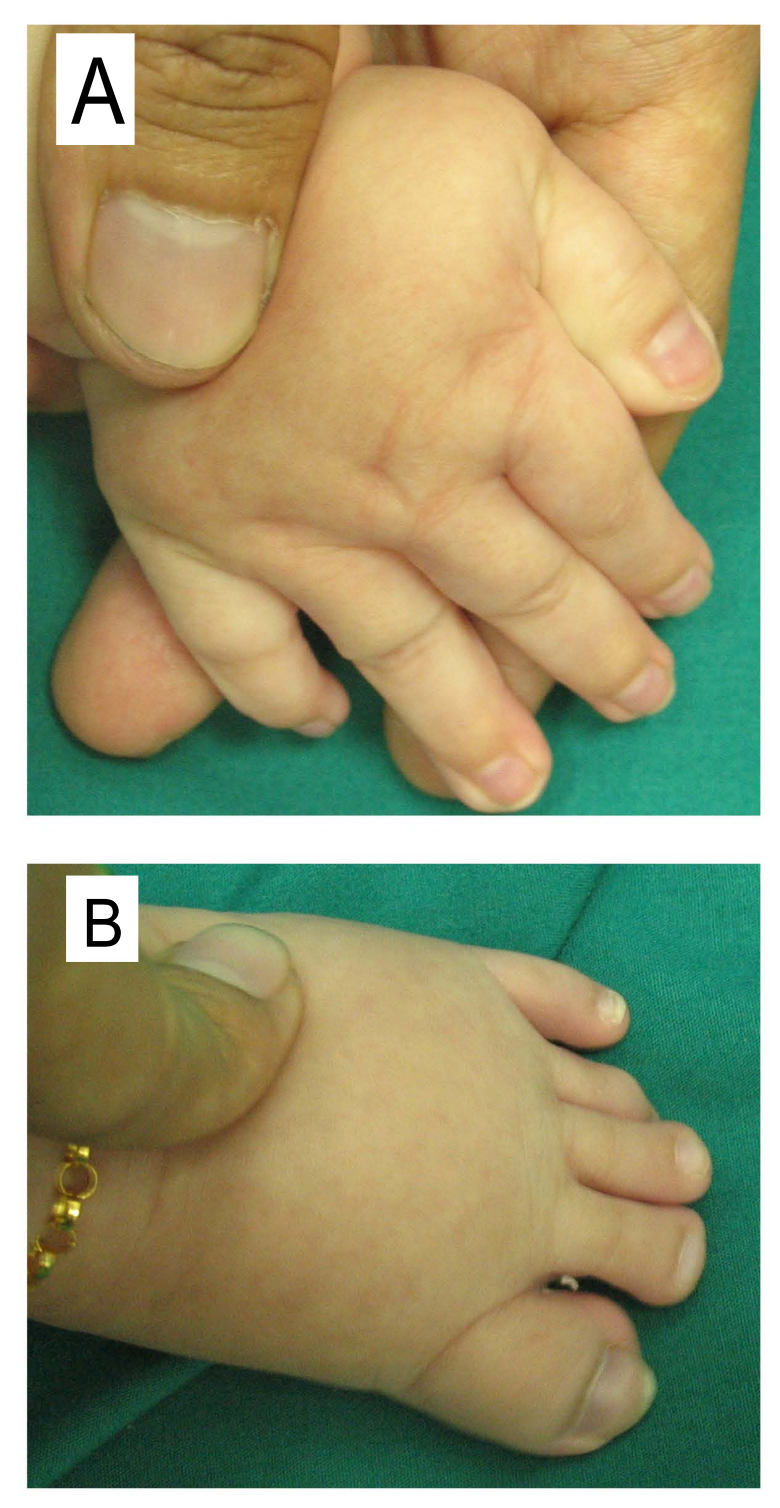
**Clinical features of our patient at the age of three years**. Dysmorphic facial features include a prominent nasal bridge, hypertelorism and down-slanting palpebral fissures. Other features include **(a) **broad thumbs and fingers and **(b) **great toes.

Our patient was a three-year-old Malaysian Chinese girl. She was the second of two children. Her parents were healthy and non-consanguineous. There was no significant family history. She was delivered at term via an elective lower segment Cesarean section with a birth weight of 2.63 kg. A pelvic cyst was detected during the antenatal period, but this had resolved spontaneously when a repeat ultrasound was done at six weeks of age. She was noted to have inspiratory stridor and a diagnosis of laryngomalacia was made at 10 weeks of age. She showed a failure to thrive with slow feeding. Her growth parameters of weight, length and head circumference were below the third percentiles. She was given nasogastric feeding. A barium swallow showed no abnormalities. Her weight gradually improved when a percutaneous gastroenterostomy tube was inserted. A physical examination at one year of age showed inspiratory stridor and dysmorphic features, such as a prominent nasal bridge, hypertelorism, down-slanting palpebral fissures, a small mouth, micrognathia, low set ears, sparse hair and bilateral broad thumbs and great toes (Figure 1). There was a soft systolic murmur heard on auscultation of the left sternal edge. She had generalized hypotonia but no other neurological deficits. She had mild to moderate global developmental delay, was only able to scribble, climb steps slowly and hold on to the rails, had no meaningful word in her speech but was able to obey simple commands and communicate with sign language. Investigations showed that she had a normal full blood count and metabolic studies, a normal karyotype and normal results for a DNA methylation study for Prader-Willi syndrome. An echocardiogram showed a moderate size atrial septal defect. She had refractive errors and failed a distraction test. A peripheral blood sample was sent for array-CGH analysis at the age of one year and seven months.

Cytogenetic analyses of our patient and her healthy parents were performed by G-banding techniques at 550 bands of resolution on metaphase chromosomes obtained by standard procedures from peripheral blood lymphocytes. Molecular karyotyping was performed using commercially available high resolution 244K 60-mer oligonucleotide microarray slide (Human Genome CGH Microarray 244A Kit, Agilent Technologies, Santa Clara, CA, USA) according to the manufacturer's protocol. This platform allows a genome-wide survey and molecular profiling of genomic aberrations with an average resolution of about 10 kb.

Cytogenetic analysis of our patient and her parents showed that they had normal karyotypes. Array-CGH analysis revealed a microdeletion of chromosome 16 involving band p13.3, with the first clone locating at 3, 651, 083 base pairs on proximal 16 p13.3 and the last clone locating at 3, 771, 464 base pairs on distal 16 p13.3, according to the University of California Santa Cruz Genome Browser on Human March 2006 Assembly (NCBI36/hg18). The size of the deletion was estimated to be about 120 kb. The deletion was confirmed by a dye swap experiment of array-CGH. Multiplex ligation-dependent probe amplification (MLPA) experiments were performed to validate the array-CGH finding and to determine the parental origin of the deletion. The deletion was confirmed to be hemizygous and *de novo *using SALSA MLPA kit P313-A1 CREBBP (Microbiology Research Centre Holland, Amsterdam, The Netherlands).

## Discussion

**Figure 2:**
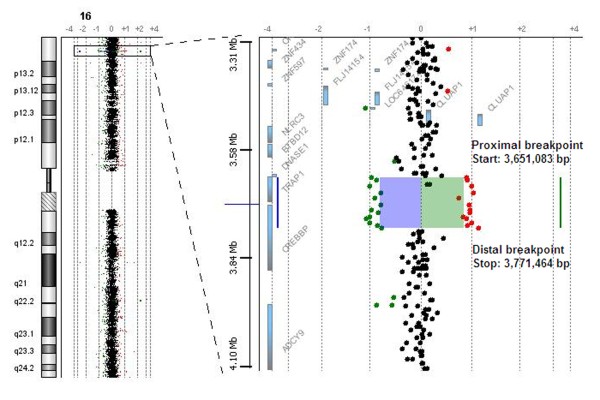
**Profile of the microarray analysis showing the deletion region as indicated in the highlighted area**. A dye-swap experiment was performed to maximize the accuracy of detection of the deletion.

**Figure 3:**
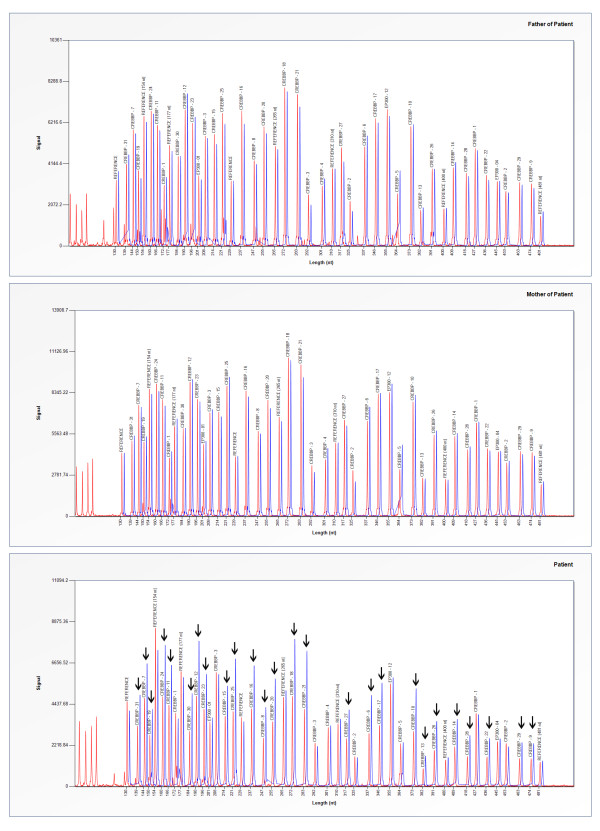
**The MLPA profile demonstrating a *de novo *hemizygous deletion of exons 6 to 31 of the *CREBBP *gene as indicated by the arrow**.

Chromosomal imbalances are known causes of genetic disorders and often result in a syndromic condition. We here describe a three-year-old girl with a global developmental delay, dysmorphic features and multiple congenital anomalies, carrying a 120 kb microdeletion of chromosome 16 p13.3 detected by array-CGH (Figure 2). The deletion region encompasses two known genes, for tumor necrosis factor receptor-associated protein 1 (*Trap1*) and *Crebbp*. It has been demonstrated that the *Trap1 *gene is located on chromosome 16 p13.3 between 3, 648, 039 and 3, 707, 599 base pairs while the *Crebbp *gene is located on chromosome 16 p13.3 between 3, 716, 570 and 3, 870, 712 base pairs (NCBI36/hg18). The deleted region in our patient, identified by array-CGH, was located between 3, 651, 083 and 3, 771, 464 base pairs. *Crebbp *was partially deleted while its neighbor gene, *Trap1*, was deleted. Further study by MLPA technique confirmed the deletion within the *Crebbp *gene to be *de novo *and hemizygous from exons 6 to 31, which corresponds to between 3, 717, 675 and 3, 772, 856 base pairs (Figure 3). This MLPA finding is consistent with the array-CGH result.

In most cases, cytogenetic rearrangements at chromosome 16 p13.3 or submicroscopic deletions within 16 p13 were found to be caused by heterozygous molecular mutations in *Crebbp *gene [4,5]. The *Crebbp *gene has been reported to be associated with RSTS and is thought to be responsible for the core clinical manifestations of RSTS in our patient. *Crebbp *(OMIM: 600140) is a transcription coactivator and functions as a potent histone acetyltransferase, both of which are essential to normal development [6]. The *Crebbp *gene is involved in different signaling pathways and in certain cellular functions, such as DNA repair, cell growth, differentiation, apoptosis and tumor suppression. *Crebbp *gene deletion [7] or exon deletion [8] are demonstrated in about 8% to 12% of patients. *EP300 *(OMIM: 602700) mutations were also identified as another rare cause of RSTS in 3% of patients [6]. The Human Gene Mutation Database (http://www.hgmd.org) holds, at present, 92 different mutations in the *Crebbp *gene - 13 missense substitutions, 20 nonsense substitutions, 10 splicing substitutions, 16 small deletions, nine small insertions, two small indels, 19 gross deletions, one gross insertion and two complex rearrangements. Previous studies of patients with RSTS indicated 16 p13.3 deletions of up to 560 kb to 650 kb and including the 5'- and 3'-flanking regions of the *Crebbp *gene [4,8,9]. Recently, intragenic deletions were detected in two RSTS patients using an exon coverage microarray platform; one in a male patient resulting from deletion of two exons within the *Crebbp *gene and the other in a female patient resulting from deletion of four exons within the *Ep300 *gene [10].

Trap1 (OMIM: 606237) is a mitochondrial 90-kilodalton heat shock protein (Hsp90). Hsp90 proteins are important and highly conserved molecular chaperones that have key roles in signal transduction, protein folding, protein degradation and morphologic evolution. Hsp90 proteins normally associate with other co-chaperones and play important roles in folding newly synthesized proteins or stabilizing and refolding denatured proteins after stress. The human Hsp90 family includes 17 genes that fall into four classes. Six genes, *Hsp90AA1, Hsp90AA2*, *Hsp90N*, *Hsp90AB1*, *Hsp90B1 *and *Trap1*, were recognized as functional, and the remaining 11 genes were considered putative pseudogenes [11]. Although *Trap1 *has not been associated with human disease, the importance of this gene warrants attention. In addition, a small subset of RSTS cases caused by 16 p13.3 microdeletions involving neighboring genes of *Crebbp *have recently been suggested to be a true contiguous gene syndrome called severe RSTS, or 16 p13.3 deletion syndrome (OMIM: 610543) [12]. Bartsch *et al*. [12] concluded that contiguous gene deletions up to 3 Mb, all of which included *Crebbp, Trap1 *and deoxyribonuclease 1 gene (*DNase1*), will result in severe RSTS. A case described by Wójcik *et al*. [13] showed an approximate 520.7 kb microdeletion on 16 p13.3, involving *Crebbp*, the Adenyl cyclase 9 and Sarcalumenin genes, in a two-year-old female with RSTS. Besides most of the typical features of RSTS, their patient had corpus callosum dysgenesis and a Chiari type I malformation which required neurosurgical decompression. These evidences demonstrated that microdeletion involving neighboring genes to *Crebbp *gene might be responsible for the additional features in RSTS. In addition, our finding merits further interest because no cases of microdeletion 16 p13.3 with contiguous gene deletion, comprising *Crebbp *and *Trap1 *genes, have been described in typical RSTS patients.

It is noteworthy that as our patient grew older her clinical features became increasingly more consistent with RSTS. Most of the typical RSTS features as described by Hennekam [3] were observed. These features include a prominent nasal bridge, down-slanting palpebral fissures, a small mouth, low set ears, bilateral broad thumbs and great toes and growth retardation. Congenital heart defects (mainly patent ductus arteriosus, ventricular septal defect and atrial septal defect) were observed in 32% of patients [3]. Although excessive hair growth (hirsutism) is found in 75% of cases [14], our patient interestingly had sparse hair, a condition that has not previously been reported in a typical RSTS patient. Whether this manifestation is affected by the abnormality of *Trap1 *is uncertain. Since our patient showed most of the typical features of RSTS, we hypothesized that this region is haplosufficient inasmuch that the deletion of *Trap1 *bore no significant complication to our patient. However, further studies are needed to clarify the potential role of *Trap1 *in 16 p13.3 microdeletion.

RSTS can be regarded as a microdeletion syndrome with a low rate of microdeletions, as only 4% to 25% of patients with RSTS were found to have the *Crebbp *deletion on chromosome 16 p13.3 when using fluorescence *in situ *hybridization [7]. It is critical that the diagnosis of an unexplained syndromic condition, especially in young patients, is confirmed by an advanced laboratory technique such as array-CGH. An accurate diagnosis will enable the precision of long-term care and a health surveillance program for patients with RSTS. This includes regular monitoring for the emergence of tumors, immunodeficiencies, early surgical correction of surgical and orthopedic complications, genetic counseling for future pregnancy and prenatal diagnosis. In addition, there is little published literature of longitudinal studies on this syndrome in the population. The finding from our patient adds to the increased awareness of RSTS variability in the population. Our case has been deposited into the Database of Chromosomal Imbalance and Phenotype in Humans using Ensembl Resources (DECIPHER-Patient ID: 254034) [15].

## Conclusion

We found this case worthy of reporting as, to date, there is very little documented evidence of this syndrome ascertained by microarray analysis. Here, we demonstrate the superiority of the array-CGH technique over conventional techniques in the screening and detection of clinically significant genomic imbalances in the human genome. Array-CGH is a reliable and powerful tool in the investigation of rare genetic conditions, especially cases with a highly variable phenotype and variable aberrations such as Rubinstein-Taybi syndrome.

## Consent

Written informed consent was obtained from the parents of our patient for publication of this case report and any accompanying images. A copy of the written consent is available for review by the Editor-in-Chief of this journal.

## Competing interests

The authors declare that they have no competing interests.

## Authors' contributions

MFMA conducted the array CGH work, performed the data analysis relevant to this case report and drafted this manuscript. TMK provided all clinical details and genetic counseling for the patient and her parents. AI performed the checking procedures for this case and reviewed the manuscript. TMK, NMY, ZZ reviewed the manuscript. All authors read and approved the final manuscript.
